# Simultaneous Hydrostatic and Compressive Loading System for Mimicking the Mechanical Environment of Living Cartilage Tissue

**DOI:** 10.3390/mi14081632

**Published:** 2023-08-18

**Authors:** Minki Chang, Yosuke Takahashi, Kyosuke Miyahira, Yuma Omuro, Kevin Montagne, Ryusei Yamada, Junki Gondo, Yu Kambe, Masashi Yasuno, Noriyasu Masumoto, Takashi Ushida, Katsuko S. Furukawa

**Affiliations:** 1 Department of Bioengineering, Graduate School of Engineering, University of Tokyo, Tokyo 113-8656, Japan; chang@biomed.t.u-tokyo.ac.jp (M.C.); yumabuma410@gmail.com (Y.O.); 2 Department of Mechanical Engineering, Graduate School of Engineering, University of Tokyo, Tokyo 113-8656, Japan; bomber.yosuke@gmail.com (Y.T.); kyomiyah777@yahoo.co.jp (K.M.); montagne@m.u-tokyo.ac.jp (K.M.); ryusei19980823@gmail.com (R.Y.); atsukona1209@gmail.com (J.G.); kambe-yu789@g.ecc.u-tokyo.ac.jp (Y.K.); ushida@mech.t.u-tokyo.ac.jp (T.U.); 3 Department of Mechanical Engineering, Faculty of Fundamental Engineering, Nippon Institute of Technology, Saitama 345-8501, Japan; d.l.finicell.t@gmail.com (M.Y.); masumoto@nit.ac.jp (N.M.)

**Keywords:** articular cartilage, mechanical stimulation, bioreactor

## Abstract

In vivo, articular cartilage tissue is surrounded by a cartilage membrane, and hydrostatic pressure (HP) and compressive strain increase simultaneously with the compressive stress. However, it has been impossible to investigate the effects of simultaneous loading in vitro. In this study, a bioreactor capable of applying compressive stress under HP was developed to reproduce ex vivo the same physical loading environment found in cartilage. First, a HP stimulation unit was constructed to apply a cyclic HP pressure-resistant chamber by controlling a pump and valve. A compression-loading mechanism that can apply compressive stress using an electromagnetic force was implemented in the chamber. The synchronization between the compression and HP units was evaluated, and the stimulation parameters were quantitatively evaluated. Physiological HP and compressive strain were applied to the chondrocytes encapsulated in alginate and gelatin gels after applying high HP at 25 MPa, which induced damage to the chondrocytes. It was found that compressive stimulation increased the expression of genes related to osteoarthritis. Furthermore, the simultaneous application of compressive strain and HP, which is similar to the physiological environment in cartilage, had an inhibitory effect on the expression of genes related to osteoarthritis. HP alone also suppressed the expression of osteoarthritis-related genes. Therefore, the simultaneous hydrostatic and compressive stress-loading device developed to simulate the mechanical environment in vivo may be an important tool for elucidating the mechanisms of disease onset and homeostasis in cartilage.

## 1. Introduction

The chondrocytes in articular cartilage are highly affected by mechanical stimuli. Articular cartilage (AC) is exposed to hydrostatic pressure (HP), compressive, and shear stresses in vivo [1]. These mechanical stimuli are generated by flexion and extension movements and loading of the knee joint during daily activities, and the order of the mechanical stimuli depends on the intensity of the activity [2,3]. Articular cartilage has a water content of up to 80% owing to the fiber structure of the extracellular matrix (mostly proteoglycans and collagen). HP is simultaneously applied during compression of cartilage tissue owing to weight loading [4]. HP and compressive stress are mechanical stimuli that are sensed by chondrocytes at any position in the AC and are very important for the activity of chondrocytes [5]. In contrast, shear stress occurs mainly in the superficial layers of the AC and modulates the activity of chondrocytes in that area [6].

Physiological mechanical stimulation plays a very important role in the developmental process of osteochondral tissue through endochondral ossification [7,8] and in cellular homeostatic functions such as matrix production, differentiation, and proliferation [9]. Chondrocytes isolated from native AC and cultured in monolayers undergo dedifferentiation, as shown by a decreased production of extracellular matrix [10,11], but their function can be restored after mechanical loading [12,13]. In addition, some studies have demonstrated the possibility of inducing differentiation into chondrocytes by applying mechanical stimulation to stem cells [14,15]. On the other hand, non-physiological mechanical stimuli, especially excessive stimulation, cause tissue degradation and degeneration of chondrocytes and have been speculated to be the cause of joint diseases such as osteoarthritis [16,17]. Thus, elucidating the effects of mechanical stimuli on the activity of chondrocytes, which exist in an environment where HP, compression, and shear stress are routinely present, is very important not only for elucidating the basic functions of chondrocytes but also for clarifying pathologies such as arthritis and for potential treatment and cartilage regeneration.

So far, several types of bioreactors have been developed to reproduce the mechanical stimulation environment of the knee joint. Freeman et al. applied cyclic HP to aggregated human MSCs using a device fabricated with a pressure-resistant chamber and a piston pump and showed that differentiation into chondrocytes and osteogenesis were promoted in the presence of osteogenic factors [18]. Nazemport et al. designed an apparatus with a chamber capable of perfusing medium and subjected bovine chondrocyte pellets to oscillating HP, which significantly increased the synthesis of glycosaminoglycans and collagen, the main components of the extracellular matrix (ECM) [19]. In addition, Vainieri et al. developed a mechanism that can simultaneously apply compressive and shear stresses and applied combined stimulation to specimens harvested from bovine cartilage tissue, reporting an increase in ECM production [20]. Park et al. also developed a combined shear and compression loading device with a mechanism different from that of Vainieri et al. and showed that combined mechanical stimulation not only increased ECM production but also induced different cell orientations in different layers [21]. A variety of studies have reported bioreactor designs that can reproduce each mechanical stimulus [22]. However, no bioreactor has realized combined compression and hydrostatic loading, which affect almost all chondrocytes in the articular cartilage. Although the effects of individual mechanical stimuli and combinations of mechanical stimuli have gradually become clear, it will be difficult to make further progress in chondrocyte research until the combined effects of major mechanical stimuli can be validated.

A major reason why the development of a bioreactor capable of simultaneously loading compressive stress and HP is considered difficult is the mainstream method of implementing each in the hardware. Compression devices generally include actuators with wiring, such as motors and load cells, and it is difficult to use these devices in a culture medium or high-pressure environment. On the other hand, HP is mostly applied by a mechanism that loads HP by pressurizing a sealed chamber filled with water or culture medium [13]. Therefore, it has been difficult to combine these two types of mechanical stimulation.

In vivo, the articular cartilage tissue is covered with a cartilage membrane and has a high-water storage capacity. Therefore, HP is thought to be induced by compressive stress loading. However, gels that are often used for 3D culture of chondrocytes in vitro are unlikely to generate HP by compressive loading because there is no external membrane and their water retention capacity is poor [13,23]. Therefore, the purpose of this study was to reproduce the physical environment of cartilage tissue in vivo by developing a prototype device that can simultaneously and independently control compression and HP.

## 2. Materials and Methods

### 2.1. Required Functions of Bioreactor

The following required functions were listed to realize the objective of the bioreactor: to fabricate a device capable of simultaneously applying compressive stress and HP to reproduce the in vivo environment: 1. Synchronous control of HP and compressive stress units; 2. The bioreactor can apply cyclic HP (1~10 MPa) and compressive strain (1~20%) to cell-scaffold constructs; 3. The bioreactor has the capability of using a small amount of culture medium (up to 10 mL) to reduce the running costs of experiments, especially for pharmacological inhibitor testing; 4. The temperature of the culture medium can be maintained at 37 °C or lower; 5. The area filled with medium must be a sealed space to prevent contamination; 6. All parts in contact with the culture medium must be made of sterilizable materials to prevent contamination.

### 2.2. Fabrication of Bioreactor

To design a mechanism for a compression unit that is compatible with an HP unit, consisting of a pump, chambers made of glass or plastic (non-magnetic material), a pressure sensor, and a valve, all actuators of the compression unit that were not suitable for environments in a closed chamber, high pressure, or liquid were eliminated, and an electromagnetic force that enables remote compression loading was used.

All compression parts set in the chamber were designed by 3D-CAD (Fusion360, Autodesk, San Rafael, CA, USA) and fabricated by “Agilista”, a high-definition 3D printer (KEYENCE, Osaka, Japan) and all parts in contact with cells and culture media were sterilized with ethylene oxide gas before the experiment.

The flow of the mechanical stimulus load is as follows: water pumped from the water bath circulates through the left chamber (HP loading), HP sensor and valve, and right chamber (HP loading), and then returns to the water bath. Cyclic HP was applied by controlling the opening and closing of the valves during the water circulation. The left chamber was located between the HP sensor and valve, so the sample in this chamber was subjected to cyclic HP, whereas the sample in the right chamber was not. The analog signal output from the HP sensor of the HP unit is processed by the Arduino Due, a microcontroller, and goes through an electronic circuit including a relay that can switch the direction of the current cyclically to control the magnetic field of the solenoid coil (The sketch is available upon request). The neodymium magnets contained in the compression parts set inside the syringe in the chambers move back-and-forth under a controlled magnetic field to apply a compressive load to the sample under cyclic HP.

### 2.3. Electromagnetic Force Calculation and Circuit Design

**Figure 1:**
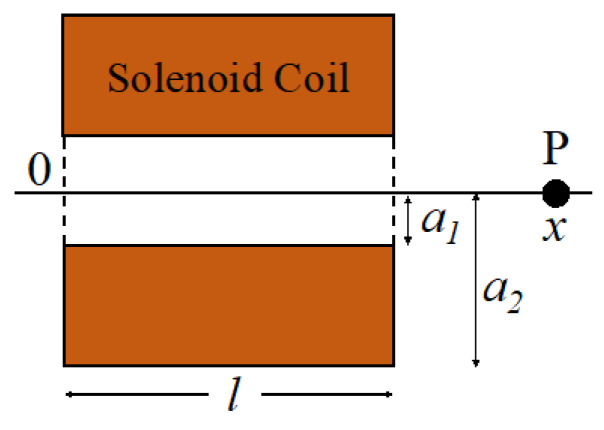
Schematic of the coil parameters. Parameters required to calculate theoretical values of the solenoid coil. “a_1_”: Inner radius (m), “a_2_”: Outer radius (m), “l”: Coil length (m), “x”: Distance (m) from “0”.

To apply cyclic compressive stress to a sample, it is necessary to control the magnetic field generated by the solenoid coil and act on a neodymium magnet installed inside the compression parts. First, theoretical calculations of the output generated by the solenoid coil and neodymium magnet were performed. The coil parameters shown in Figure 1 were used. The theoretical output can be calculated as follows:

*H*: Magnetic field strength at point P

(1) $$H=\frac{NI}{2l\left(a_{2}-a_{1}\right)}\left\{xlog\frac{a_{2}+\sqrt{a_{2}^{2}+x^{2}}}{a_{1}+\sqrt{a_{1}^{2}+x^{2}}}+(l-x)log\frac{a_{2}+\sqrt{a_{2}^{2}+{\left(l-x\right)}^{2}}}{a_{1}+\sqrt{a_{1}^{2}+{\left(l-x\right)}^{2}}}\right\}\;$$

M: Magnetic moment

(2) $$M\left(Wbm\right)=\frac{\pi R^{2}B_{r}}{2}\left(\frac{L}{\sqrt{R^{2}+L^{2}}}\right)\;$$

*R*: magnet radius (m), *L*: magnet thickness (m), and *B_r_:* remanence (mT).

*F*: Force acting on a magnet at point *P*

(3) $$\left|F\right|=\frac{\partial }{\partial x}(|M|\cdot |H|)\;$$

The compressive force of the neodymium magnet in the magnetic field generated by the solenoid coil was measured using the Magnetic Micro Testing System (Microservo, MMT-250 N, SHIMADZU, Kyoto, Japan). Theoretical and experimental values were calculated or measured for magnet thicknesses of 20, 30, and 40 mm and current values of 4, 8, and 12 A.

### 2.4. Analog Signal Processing

To synchronize the HP unit with the compression unit, an algorithm for smoothing the signal output from the HP unit and detecting extreme values was introduced. For smoothing, an analog filter (RC filter) and a digital filter (exponentially weighted average filter) were implemented. The RC filter is a simple low-pass filter consisting of a resistor R and a capacitor C. It is a smoothing method that passes low-frequency components and blocks high-frequency ones. The cutoff frequency fc was set at 0.66 Hz, which is the frequency at the functional limit of the device, and the values of *R* = 26 kΩ and *C* = 10 µF were derived using Equation (4).

(4) $$f_{c}=\frac{1}{2\pi RC}$$

The analog signal processed by the RC filter was further processed by an exponentially weighted average filter (Equation (5)) on the Arduino to remove noise: α: smoothing parameter, t: time, $x_{t}$: the value of process variable at time t, $x_{S,t}$: smoothed value at time t),

(5) $$x_{S,t}=\alpha \left\{x_{t}+\left(1-\alpha \right)x_{t-1}+\left(1-\alpha \right)x_{t-2}\ldots \right\}$$

Extreme value detection can be determined as follows (Equations (6) and (7): t: time, a: extreme value detection width, f(t): smoothed signal value at time t.

(6) $$\mathrm{Local}\mathrm{maximum}\mathrm{value}:f\left(t-2a\right)<f\left(t-a\right)>f\left(t\right)$$

(7) $$\mathrm{Local}\mathrm{minimum}\mathrm{value}:f\left(t-2a\right)>f\left(t-a\right)>f\left(t\right)$$

### 2.5. Bioreactor Evaluation

Evaluation data for bioreactors: The signals of HP loading and compressive loading were obtained as raw data by Arduino and Processing, and then the correct rate, synchronization rate, and phase difference accuracy rate were calculated using Python. The correct rate was defined as

(8) $$\frac{\left(Correct\;number\;of\;compressive\;loading\;switching\;in\;the\;number\;of\;measured\;cycles\right)}{\left(Total\;number\;of\;measured\;cycles\right)}\;$$

The synchronization rate was defined as

(9) $$\frac{\left(Average\;time\;of\;synchronization\;of\;HP\;and\;compressive\;loading\right)}{\left(Average\;time\;per\;one\;cycle\right)}\;$$

The phase difference accuracy rate was expressed as follows, using the set ideal phase difference (Ideal Gap Time) and the measured actual phase difference (Actual Gap Time) and cycles. The parameters of the phase difference of the HP device were set to π/4, π/2, and 3π/4.

(10) $$1-\frac{\left|Average\;time\;of\left\{\left(Ideal\;Gap\;Time\right)-\left(Actual\;Gap\;Time\right)\right\}\right|}{\left(Average\;time\;per\;one\;cycle\right)}\times 100\;$$

### 2.6. Cell Culture and Encapsulation in Gel Scaffold

**Figure 2:**
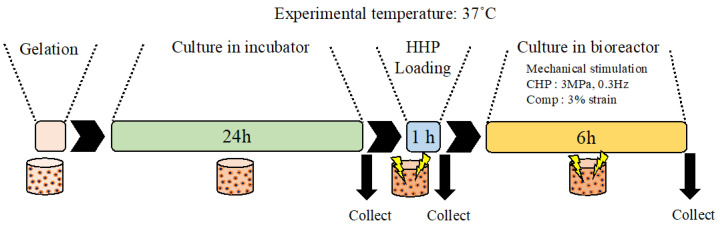
Schematic of the experimental flow. The samples were prepared using monolayer cells and gel scaffolds and incubated in an incubator for 24 h. After overstimulation of the samples with HHP at 25 MPa for 1 h to induce damage, the samples were loaded with physiological stimuli such as CHP (3 MPa, 0.3 Hz) and compression (3% strain). All cultures were conducted at 37 °C.

ATDC5 cells, a mouse chondrogenic cell line, were purchased from the Japanese Collection of Research Bioresources Cell Bank and cultured in DMEM/F12 containing 5% FBS, 4 mM of l-glutamine, and antibiotics in an incubator set at 100% humidity, 5% CO_2_, and 37 °C. The cultural medium was changed every two days. Sodium alginate (Sigma, Burlington, MA, USA) and gelatin (Sigma) were dissolved in PBS (–) to prepare 3% alginate and 15% gelatin solutions, respectively. A 2.5% alginate + 2.5% gelatin solution was prepared by mixing 3% alginate solution and 15% gelatin solution at a ratio of 5:1. The 2.5% alginate + 2.5% gelatin solution was mixed with a solution of cells suspended in the medium at ratios of 4:1, 3:2, and 2:3, and the final concentration of the gel solution was 2% alginate + 2% gelatin/1.5% alginate + 1.5% gelatin/1% alginate + 1% gelatin. The gel solution was poured into each well of the sample holder and incubated with 102 mM CaCl_2_ for 20 min. After incubation, the sample holder was washed three times with PBS (–), immersed in the culture medium, and incubated in the incubator for 24 h until the experiment. The final cell density was 1.5 × 10^6^ cells/mL. After encapsulating the ATDC5 cells, an experiment was conducted, as shown in Figure 2.

### 2.7. Mechanical Stimulation Loading

**Figure 3:**
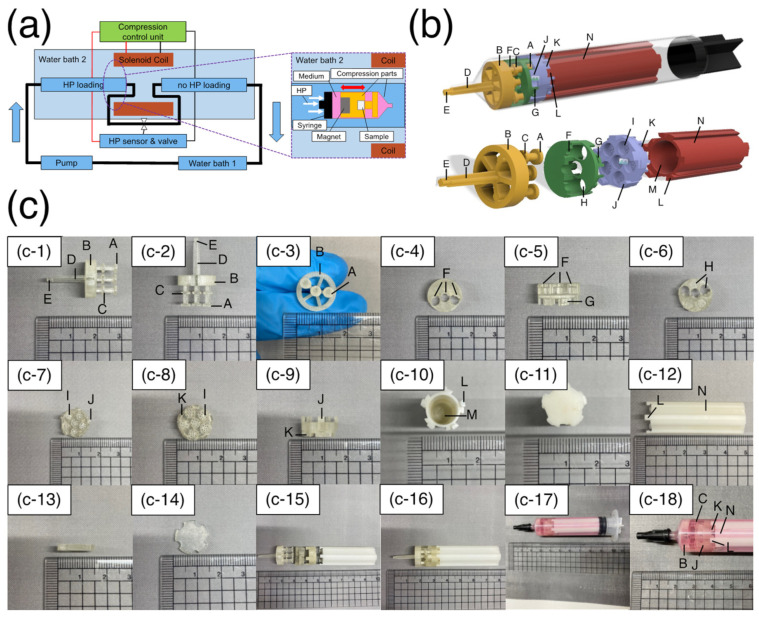
Designed bioreactor overview. (**a**) Schematic representation of the bioreactor. The HP unit is composed of water bath 1 (for water circulation in chambers), water bath 2 (for maintaining chamber temperature), a pump, chambers, an HP sensor, and a valve. The compression unit was composed of a solenoid coil, compression parts, and a control unit. (**b**) 3D-CAD modeling of compression parts. The upper panel shows the compression parts set inside the syringe. The transparent and black areas indicate the syringe rod and gasket, respectively. The lower figure shows the compression components. The yellow part indicates the indenter. A–E indicate the structures with the indenter. The green area represents the middle part. F–H indicate the structures with the middle part. The purple portion represents the sample holder. I–K indicate the structures with the sample holder. The red part represents the magnet holder. L–N indicate the structures with the magnet holder. (**c**) Images of compression parts. (**c-1**–**c-3**) Images of the fabricated indenter. (**c-4**–**c-6**) Images of the fabricated middle part. (**c-7**–**c-9**) Images of the fabricated sample holder. (**c-10**–**c-12**) Images of the fabricated magnet holder. (**c-13**,**c-14**) Images of the fabricated cover. (**c-15**,**c-16**) Images of the combination of compression components. (**c-17**,**c-18**) Images of the compression parts and samples set inside the syringe with culture medium.

**Figure 4:**
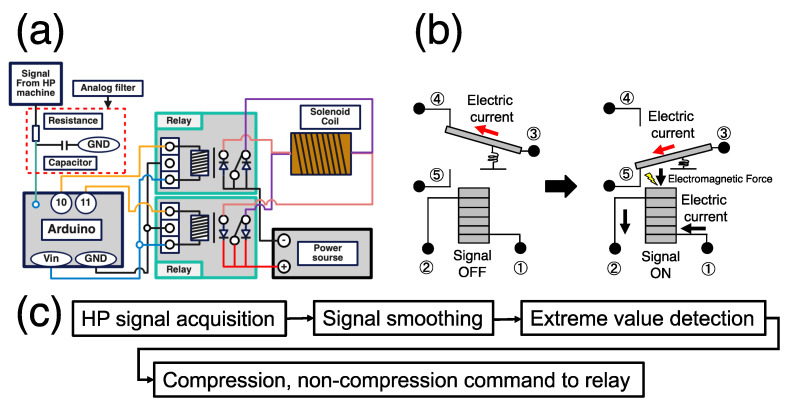
Schematic of the compression control unit. (**a**) Electronic circuit diagram. The signal output from the HP unit is processed by the Arduino, leading to the control of the relay circuits through the pins 10, 11 for signal output and solenoid coil. (**b**) Relay circuit mechanism. When the electric current flows from 1 to 2, the electromagnetic force attracts the variable circuit and changes the electric current flow from 3 to 4 to that from 3 to 5. (**c**) Signal processing algorithm for synchronizing the compression unit to the HP unit.

**Figure 5:**
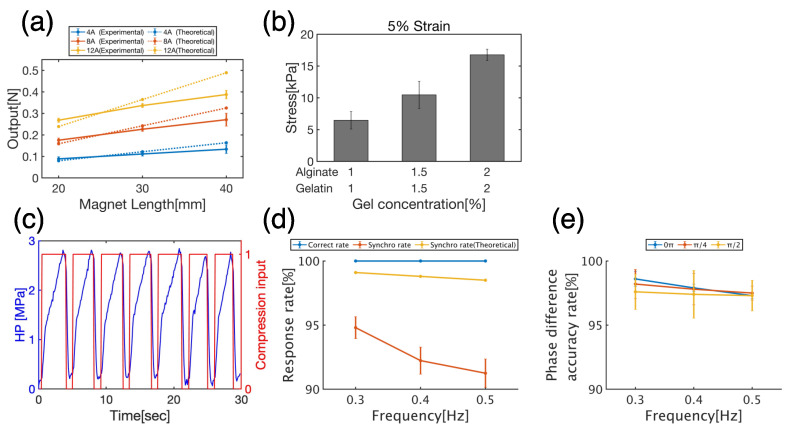
Evaluation data for bioreactor and gel scaffold. (**a**) Theoretical and experimental compressive force outputs for each magnet length and current value. The graph shows the mean ± S.E. of three independent experiments. (**b**) Mechanical properties of scaffolds at each gel concentration. (**c**) Synchronization of HP (HP, blue line) and compressive input (red line). (**d**) Response rate, indicating the synchronization of the compression signal to HP. (**e**) Phase difference accuracy rate, indicating the accuracy with respect to the set phase difference of the compression signal. The graphs (**d**,**e**) show the mean ± S.E. of the results measured in 10 independent experiments with 30 cycles per experiment.

**Figure 6:**
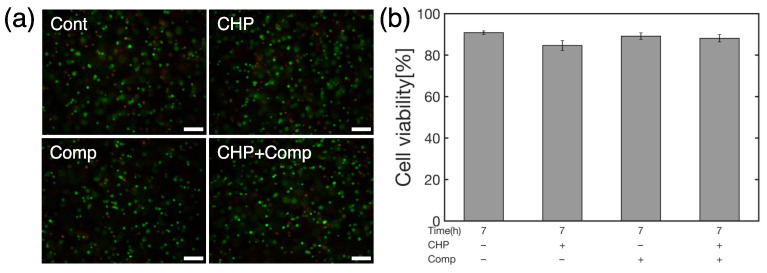
(**a**) Representative images of Calcein-AM/PI staining (scale bar = 100 µm) at 0 h. Green dots indicate live cells and red dots indicate dead cells. (**b**) Cell viability rate in control, CHP, Comp, and CHP+Comp groups (n = 4, mean ± S.E.).

**Figure 7:**
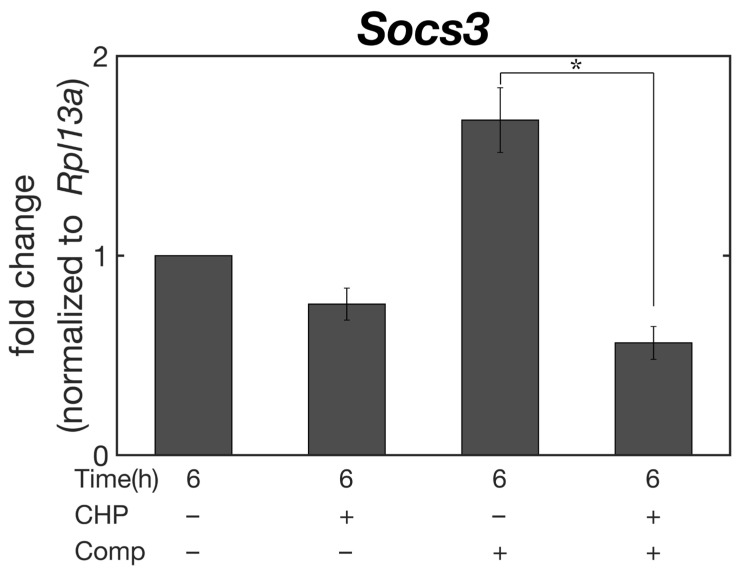
Real-time PCR analysis. Gene expression of *Socs3* in ATDC5 cells at control, CHP, Comp, and CHP+Comp (n = 4, mean ± S.E.). Gene expression was normalized to that of *Rpl13a* and the control. Asterisk indicates statistically significant difference (* *p* < 0.05).

After 24 h of static incubation, the sample holders containing samples in each well were sealed with 7 mL of culture medium in polyethylene bags (Seisan Nippon Sha Ltd., Fukuoka, Japan) and placed in the pressure chamber of the high HP loading device used in previous studies to apply 25 MPa for 1 h to induce damage to ATDC5 cells, as performed in a previous study [24]. The sample holders containing the sample were removed from the pressure chamber, combined with other compression parts, sealed in a syringe (TERUMO, Tokyo, Japan) with 7 mL of culture medium, and placed in the chamber of the HP device. The chambers containing the samples were placed in water bath 2, maintained at 37 °C. The solenoid coil was also placed in water bath 2 with waterproofing (Figure 3a). The loading parameters for the mechanical stimuli were HP of 3 MPa, compression equivalent to 3% strain, and a frequency of 0.3 Hz. One sample holder with six wells (Figure 3c(c-7)) was placed per chamber. Within the same sample holder, three wells were subjected to compressive strain, whereas the other three were not. Therefore, a control group, HP alone, compression alone, and a combination of HP and compression could be performed simultaneously in a single experiment.

### 2.8. Mechanical Testing

Samples were prepared using a sample holder, and the mechanical properties of confined compression were measured using a magnetic micro-testing system (Microservo, MMT-250N, SHIMADZU, Kyoto, Japan). Stresses were measured at 5% strain, which was within the linear compression range for each gel scaffold concentration.

### 2.9. Real-Time PCR

Total RNA was extracted from ATDC5 cells after isolation from a gel scaffold using TRIzol reagent (Invitrogen). Extracted RNA was reverse-transcribed using the ReverTra Ace qPCR RT Master Mix with gDNA Remover (Toyobo, Osaka, Japan). Real-time PCR was performed using the Thunderbird SYBR qPCR Mix (Toyobo) in a Step One Plus real-time PCR system (Life Technologies, Carlsbad, CA, USA). The primers used in this study were as follows: *Rpl13a*, forward: 5′-TCTGGAGGAGAAACGGAAGGA-3′, reverse: 5′-GGTTCACACCAAGAGTCCATTG-3′; *Socs3*, forward: 5′-AGATGGAGGGTTCTGCTTTG-3′, reverse: 5′-TGTGTTTGGCTCCTTGTGTG-3′.

### 2.10. Statistical Analysis

All data were statistically analyzed using RStudio 2022.07.2+576 (Posit, Boston, MA, USA). Before performing statistical tests, a normality test was performed. As a result, the Dunnett test was used to compare control and experimental groups, and the Tukey–Kramer test was used for multiple comparisons among experimental groups. Statistical significance was set at *p* < 0.05.

## 3. Results

### 3.1. Compression Unit

The compression unit consists of four parts: an indenter, a middle part, a sample holder, and a magnet holder. Each part had a structure that could be disassembled or combined for culture, mechanical loading, and each situation (Figure 3c(c-15)). The indenter was designed to be fixed to the tip of a 10 mL syringe (TERUMO, Tokyo, Japan) (Figure 3c(c-18)). The middle part, sample holder, and magnet holder move back and forth as one component to apply compressive stress to the samples. After the compression parts were fixed inside the syringe, a syringe cap (TERUMO, Japan) was attached to seal the syringe. Because the gasket part of the syringe was movable, when HP was applied to the chamber, the same pressure was applied to the samples inside the syringe, owing to an equilibrium effect. These mechanisms result in both hydrostatic and compressive stress. The following sections describe each component in detail.

The indenter (Figure 3b, yellow, Figure 3c(c-1–c-3)): “A” is the part that contacts and compresses the sample. A pillar and a structure with a large diameter existed at the tip (“A,” φ3.8) and in the middle (“C,” φ3.6) of the pillar. “C” of the indenter and the groove structure in the middle part (“F”, Figure 3b, green, Figure 3c(c-4–c-6)) allow a mechanism to slide a fixed distance. There are three pillar structures with “A” and “C” structures, and three of the six samples in the sample holder can be compressed simultaneously. This enabled the user to perform an experiment under multiple conditions in one sample holder. “B” is the structure that fits inside the syringe to stabilize the compression parts in the syringe. “C” is the stopper structure that limits the stroke of the indenter and prevents it from applying a hammering force to the impact surface. “D” is the slope structure that tapers toward the tip and can be fixed to the tip of the syringe. “E” is the part that protrudes from the tip of the syringe. When the indenter connected to other parts is fixed to the syringe, it is possible to pull it with a tweezer to facilitate the operation. After an experiment, “D” is pushed in for easy removal of the assembled compression parts from the syringe.

The middle part (Figure 3b, green, Figure 3c(c-4–c-6)): “F” serves as a slider for the back-and-forth motion of the indenter as it compresses the samples. “H” is the structure that distinguishes between samples that are loaded by compressive stress and samples that are not loaded within the same sample holder. “H” is designed to pass through the three pillars of the indenter. “G” is a groove structure that exists on the sides and bottom to combine with “J” of the sample holder. 

Sample holder (Figure 3b, purple, Figure 3c(c-7–c9)): This is a mold that can be used to prepare samples using a gel scaffold and can be combined with other compression parts without removing the sample after preparation. “I” is the structure designed to hold samples (diameter: φ4 m height: 3 mm). There were many 450 µm × 450 µm square pores at the bottom of each well to enable the supply of culture medium from the bottom. A thin membrane of the same diameter was placed at the bottom before the gel was poured to prevent the sample from being extruded through the pore structure during compressive loading. “J” is a pillar structure connected to the middle part. The five pillars were placed at equal intervals. The upper part of “J” has a protruding structure that can be inserted into the “G” of the middle part and rotated to fix it in place by fitting. “K” is the groove structure similar to “G” at the middle part.

Magnet holder (Figure 3b, red, Figure 3c(c-10–c-12)): this is a structure for holding magnets, in which two to four neodymium magnets of φ4 × 10 mm with a surface magnetic flux density of 491 mT are inserted. “L” is a pillar structure similar to that of the sample holder “J.” “M” is the magnet holding section and has a hollow structure that holds a φ4 mm × 40 mm magnet. “N” is a groove structure on the side of the magnet holder, designed to facilitate the circulation of culture medium inside the syringe.

Weight (Figure 3c(c-13,c-14)): the weight was placed on top of the sample holder when the sample was prepared in the sample holder. A membrane was placed between the surface of the gel carrier and the weight to allow penetration of the gel curing solution and flatten the surface of the specimen during the curing process.

Assembly (Figure 3b, upper, Figure 3c(c-16–c-18)): all compression parts were combined in the following order from the tip of the syringe: indenter, middle part, sample holder, and magnet holder. The assembled compression parts were sealed in a syringe with culture medium, and the syringe was sealed with a syringe cap. After sealing, the syringe was placed in a glass chamber for mechanical loading (Figure 3a, enlarged window).

### 3.2. Electronic Circuits

Electronic circuits were assembled to synchronize the HP and compression units (Figure 4a), and the analog signal output from the HP unit was processed. The analog signal was passed through an analog filter consisting of a resistor and capacitor, smoothed by a digital filter implemented in the Arduino Due, and extreme values were detected. The detected minimum and maximum values correspond to the compression and non-compression commands, respectively (Figure 4c). By repeating this “compression → non-compression → compression” operation, it is possible to synchronize the compression unit with the HP unit and apply cyclic stimulation. Two relays were used to achieve this operation in the electronic circuits. The relay is a component that controls the direction of one current flow by turning ON and OFF the current flow in an internal coil switch (Figure 4b). When current flows through the coil, a magnetic field is generated, attracting the switch connected to one of the branch channels and allowing the current to flow in the other channel. This mechanism enables control of the direction of the current flow. To prevent the circuits from generating heat due to the surge voltage generated when switching the direction of the current flow in the solenoid coil, a surge-absorbing diode was incorporated into the circuits.

### 3.3. Bioreactor Evaluation

The compressive force measured by the solenoid coil and neodymium magnet yielded a minimum value of 0.09 N (magnet thickness: 20 mm, current value: 4 A) and a maximum value of 0.39 N (magnet thickness: 40 mm, current value: 12 A) (Figure 5a). This value was converted to compressive stress considering the indenter diameter (1.9 mm) and number of compressed samples (3), resulting in 2.64~11.46 kPa. Based on the results of gel mechanical properties, this compressive stress has an output that can distort 1% Alginate + 1% Gelatin (6.47 ± 1.38) and 1.5% Alginate + 1.5% Gelatin (10.46 ± 2.14) gel carriers by approximately 5% (Figure 5b). For the 2% Alginate + 2% Gelatin gel scaffold, the maximum output could distort the gel carrier by approximately 3.4%.

Although the measured compressive force was relatively close to the theoretical values for the 20 mm thick magnet, the larger the magnet thickness and current, the larger the discrepancy between the theoretical and measured values. This may be because the model used to derive Equation (2) becomes inapplicable as the magnet length increases. The model used to derive Equation (2) is a permanent-magnet design method called the charge model. However, this method can only be applied when the magnetic poles are uniformly distributed and may not be applicable as the magnet becomes thicker. It has been found that the remanence of neodymium magnets saturates even when the thickness is increased. Therefore, it is necessary to apply other theoretical calculation models to further improve the output.

First, it was confirmed that the compression unit followed the HP load (Figure 5c). The compression unit detected extreme values in the HP signal smoothed by analog signal processing and controlled the compression unit to send a compression signal (=1) at the local maximum value and an uncompressed signal (=0) at the local minimum value. Next, to evaluate the synchronization performance of the compression unit with respect to the HP unit, the correct rate, synchronization rate, and phase difference accuracy rate defined by Equations (8), (9), and (10), respectively, were measured. 

No false positives were detected at any of the measured frequencies (0.3, 0.4, or 0.5 Hz), and a 100% correct rate was achieved (Figure 5d, blue line). Because frequencies in this range are frequently used in stimulation-loaded cultures of chondrocytes, there is no problem with determining the correct rate in culture experiments. The theoretical synchronization rate, calculated as the time delay required for signal processing by the Arduino, was 97–99% (Figure 5d, yellow line), but the actual measured synchronization rate was 92–95% (Figure 5d, orange line). This was due to the additional delay in the time required for analog signal processing from the HP unit.

The accuracy rate of the phase difference was 95–99% for all cycles (Figure 5e). This implies that there was a delay of up to 5% relative to the set phase difference. Both the synchronization rate and phase-difference accuracy rate decreased as the frequency increased. This may be due to the parameter settings in the smoothing and extreme value algorithms.

The current algorithm uses fixed parameters rather than variable parameters for each frequency. To achieve a constant synchronization rate and phase-difference accuracy rate regardless of the frequency, it is necessary to search for the optimal parameters for each frequency.

### 3.4. Tissue Culture and Gene Expressison Analysis

As a model for mechanically induced cell damage similar to that found in osteoarthritis [24], mouse ATDC5 chondrocyte progenitor cells were subjected to HP at 25 MPa for 1 h and then to mechanical stimuli that mimicked the mechanical environment of cartilage tissue during recovery (Figure 2). We analyzed cell viability (Figure 6) and the expression levels of genes related to osteoarthritis (Figure 7) after 6 h of HP only (CHP), compressive strain only (Comp), and combined hydrostatic and compressive strain only (CHP+Comp) stimulation (Figure 7). The cells were incubated for 6 h after being subjected to a HP of 25 MPa for 1 h as a trigger for the onset of degenerative arthritis, and the cells were determined to be alive or dead. The staining results are shown in Figure 6a, where live cells are stained green and dead cells are stained red. A quantitative analysis of the cell viability is shown in Figure 6b. The results showed high survival rates for static, hydrostatic-only (CHP), compression-only (Comp), and combined compression and hydrostatic stimulation (CHP+Comp), with or without stimulation.

*Socs3*, a gene associated with the development of osteoarthritis [25], was suppressed by hydrostatic-only stimulation (CHP), which is the physical environment of normal cartilage tissue in non-weight-bearing areas. Similarly, in simultaneous hydrostatic and compressive strain (CHP+Comp), which is the mechanical environment of cartilage in the weight-bearing area, *Socs3* gene expression was also suppressed in culture under simultaneous HP and compressive strain (CHP+Comp). In contrast, a significant increase in *Socs3* expression was observed in the physical environment of compressive strain only (Comp), which is considered to exist in pathological cartilage tissue, and a statistically significant difference in *Socs3* expression was observed between the physiological physical environment of compressive strain and HP simultaneously applied to cartilage tissue.

## 4. Discussion

In this study, we developed a bioreactor that can simultaneously apply HP and compressive strain, which are the main mechanical stimuli sensed by chondrocytes in vivo. We evaluated the performance of the device in terms of power output, synchronization performance, and the possibility of cell culture.

The HP unit of the developed bioreactor consists of a pump, piping, valves, and pressure-resistant chambers, which have the same mechanism as the existing bioreactors for HP loading. However, the compression unit of our bioreactor generates compressive strain via an electromagnetic force, which is a completely different mechanism from that of existing devices. Therefore, this bioreactor is highly original in that it achieves both HP and compressive strain, which have not been achieved before. Many studies have been conducted to verify the effects of HP, or compressive strain, on chondrocytes. However, examining the effects of individual mechanical stimulation is not sufficient to collect information on the mechanosensitivity of chondrocytes.

Therefore, the study of the mechanosensitivity of chondrocytes using this bioreactor can reveal the single and combined effects of HP, which is an isotropic stimulus for all molecules of the cell from the plasma membrane to the cytoplasm and nucleus, and compressive stimuli, which cause cell deformation and partially affect cellular molecules such as the plasma membrane and cytoskeleton. This will advance our understanding of the critical role of the mechanosensitivity of chondrocytes, which will be useful not only for understanding simple biological phenomena but also for applications such as the creation of regenerated cartilage tissue and the development of treatments for articular cartilage-related diseases.

The parameters that can be applied by this device are HP of up to 6 MPa, compressive strain of up to approximately 5% (the concentration of the gel carrier must be adjusted), and frequency of 0.3~0.5 Hz. In general, the parameters used to investigate the physiological activity of chondrocytes and prepare regenerated cartilage are HP in the range of 1–10 MPa, compressive strain in the range of 1–20%, and frequency in the range of 0.1~1 Hz [13]. Although the parameters were within the range of existing studies, further improvement is needed to apply a larger mechanical stimulus. Because this bioreactor uses glass chambers for HP, which are less durable than metal, it is necessary to use high-pressure-resistant materials such as sapphire glass to increase the loading parameter of the HP. In addition, the compressive strain applied to the sample holder of this bioreactor was due to confined compression; therefore, a very high compressive force was required as the amount of desired strain increased. For research on regenerated cartilage, it is necessary to use a material with high mechanical properties; therefore, it is essential to increase the compressive force. To achieve this, the magnetic field can be strengthened or the thickness of the magnet can be changed, but the former method is more realistic given the limitations of the narrow space inside the syringe. To strengthen the magnetic field, the number of solenoid coils should be increased, and a higher electric current should be applied. In addition, circuit design and cooling systems to suppress the heat generated by the high current are necessary.

In addition to the output, this bioreactor has several limitations and disadvantages. The most significant limitation of this study was its narrow research area. With the mechanical stimulation parameters of the bioreactor and the culture system in a syringe, research is limited to analyzing short-term cellular responses for a few hours or more. This is because this bioreactor can apply physiological compression stimuli only when HP is within the physiological range and a gel scaffold with low mechanical properties is used, and because the compression parts, including samples and culture medium, are simultaneously enclosed in a syringe, there is a high risk of contamination when the medium is changed. By solving these limitations, it is possible to load the appropriate mechanical stimulation parameters onto chondrocytes over a long period of time, thus matching the mechanical environment in which the chondrocytes reside within the cartilage in vivo.

The other major limitation of this study is that we were not able to reproduce the extracellular matrix structure in which the chondrocytes are located. In addition to reproducing the mechanical environment, the physical environment of cartilage tissue can be realized by reproducing the extracellular tissue in which chondrocytes exist. However, fabricating structures with a composition similar to that of cartilage in vivo is also one of the most challenging research problems in the study of cartilage tissue. This is because it is necessary to verify many parameters, such as extracellular matrix density, pore structure, composition, mechanical properties, cell adhesion, and biocompatibility. Therefore, in this study, we did not examine this part of the process but focused only on reproducing the mechanical environment and employed a composite of alginate gel and gelatin, which are commonly used in the study of cartilage tissue, as the material. Future research on materials is expected to reproduce the complete physical environment of cartilage tissue. Resolving these challenges will enable the creation of regenerated cartilage tissue and research to gain insights into articular cartilage-related diseases.

In addition, it is almost impossible to achieve 100% for all parameters used to evaluate synchronization performance (correct response rate, synchronization rate, and phase difference accuracy rate) because of the delays that occur during the signal processing process. Therefore, the results of the cellular responses to combined stimuli should be considered with some delay.

We would also like to consider adding other functions. The current compression unit cannot monitor the sample compression in real time. A compression monitoring mechanism that observes the movement of the indenter using optical measurement technology will be necessary to apply a more accurate and reproducible compression stimulus. In addition, the development of the device and discussion of the experimental results should be conducted while keeping in mind that there are various reports on the effects of cells on electromagnetic fields. For example, it has been reported that pulsed electromagnetic fields demonstrate not only physiological effects, such as an increase in glycosaminoglycans and cell proliferation, but also healing effects, such as anti-inflammatory effects and inhibition of ECM degradation [26,27].

In cellular experiments, we examined the expression level of *Socs3*, which has been suggested to be associated with the onset of osteoarthritis [25]. Three types of physical stimuli, HP only, compressive strain only, and combined hydrostatic and compressive stress, were applied to a model sample [24] after subjecting the cells to excessive, damage-inducing pressure at 25 MPa. The results showed that the expression of *Socs3* was extremely low in the sample while simultaneously subjected to compressive strain and HP, which reproduced the physiological environment of the loaded area. The suppression of *Socs3* expression was also observed in the sample to which only HP was applied, which exists in the non-loaded area of normal cartilage tissues. In contrast, compression-only stimulation, a physical stimulus that can be present at sites of knee osteoarthritis where the ECM has degenerated, was found to increase *Socs3* expression. These results suggest that physical stimulation with the experimental system developed in this study can move the cartilage condition to a more favorable physiological situation as well as to pathological tissue. Therefore, it can be shown that the bioreactor developed in this study can serve as an engineering model to study the effects of the physical environment on cartilage in detail.

In this study, to understand the mechanism of knee osteoarthritis, which is a social problem with a high prevalence among the elderly, we developed a model to investigate how the healing process is affected by the physiological mechanical environment using a model in which cartilage cells are subjected to a HP of 25 MPa, which the authors have previously shown to mimic some of the gene expression changes observed in osteoarthritis. The model was created to investigate how the healing process changes depending on the physiological and mechanical environment. Although many experimental issues must be addressed biologically, such as the type of chondrocytes, the number of days of culture, and the amplitude and frequency of the physical stimuli, we developed a bioreactor from a mechanical engineering perspective and demonstrated the usefulness of the model.

## 5. Conclusions

Compressive stress and HP are the main mechanical stimuli in biological cartilage, and these forces may control the homeostasis of cartilage tissue and the development of diseases, such as osteoarthritis. The bioreactor developed in this study, which can simultaneously apply compressive stress and HP, may be an important engineering technology for elucidating these mechanisms and promoting their applications in preventive and regenerative medicine.

## Data Availability

Data are available on request from the authors.
